# Concurrent Bilateral M1 and Anodal Cerebellar tDCS Effects on Learning of a Bimanual Video Game Task

**DOI:** 10.1002/brb3.71076

**Published:** 2025-11-25

**Authors:** Quinn McCallion, Davin Greenwell, Bryan S. Barry, Emelia E. Duchow, Brach Poston, Zachary A. Riley

**Affiliations:** ^1^ School of Health and Human Sciences Indiana University‐ Indianapolis Indiana USA; ^2^ Department of Kinesiology and Nutrition Sciences University of Nevada‐Las Vegas Las Vegas Nevada USA

## Abstract

**Purpose:**

The primary motor cortex (M1) and the cerebellum are important sites of processing for motor learning of complex, bimanual tasks. However, little is known about the current and polarity effects of transcranial direct current stimulation (tDCS) when applied concurrently to these sites during learning. Therefore, the present study sought to examine the effect of bilateral primary motor cortex (M1) anodal tDCS coupled with anodal cerebellar tDCS (biM1a + CBa) on learning of a bimanual racing videogame.

**Method:**

Forty‐six subjects were enrolled and received either biM1a + CBa (*n* = 23) or biM1a + CBsham (*n* = 23) stimulation for a single practice session. Additional data from a previous study in our lab using bilateral primary motor cortex (M1) anodal tDCS coupled with cathodal cerebellar tDCS (biM1a + CBc, *n* = 21) and a SHAM condition (*n* = 20) was included in our analysis. Videogame performance was assessed before and after the practice session, and a follow‐up assessment took place 24 h later.

**Finding:**

Learning and previous gaming experience were negatively correlated, so experience was included as a covariant in the analysis of results. Consistent with the current literature, simultaneous tDCS of both motor cortices, without any cerebellar stimulation, improved learning the most within a single day (*p* = 0.025). No significant retention effects were observed for any of the conditions (*p =* 0.87).

**Conclusion:**

These results reinforce the benefits of tDCS to M1 and that, at least in the video game task used in this study, cerebellar stimulation does not add to the learning effect. When using multifocal stimulation montages, careful consideration needs to be given to the relationship between electrode placement, task specificity, previous experience, and individual differences of the participants.

## Graphical abstract

Subjects played a racing kart game while receiving several different transcranial direct current stimulation (tDCS) stimulation conditions. All subjects improved with practice of the game, but those receiving just bilateral, anodal motor cortex transcranial direct current stimulation (M1 tDCS) improved the most.

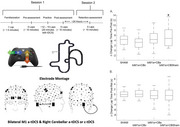

## 1 Introduction

Two‐handed tasks are an important part of interacting with our environment, either working together on the same task (e.g., tying shoelaces) or performing independent actions (e.g., moving a computer mouse and typing simultaneously). Motor learning—the progressive change in performance or execution of a motor task—is more neurologically complex in a bimanual movement (Jie et al. 2024) and requires elevated activity from areas such as the primary motor cortex (M1) and the cerebellum (Nakashima et al. 2021; Rastogi et al. 2017; Yang et al. 2021).

The primary motor cortex contains densely packed interconnections with the somatosensory cortex, the cerebellum, and other brain regions. Millions of neuronal inputs from these areas converge in M1, are integrated, and descend as efferent signals to the periphery (Rizvi et al. 2023). For instance, during the earliest stages of motor learning, neurons in the somatosensory cortex first become more excitable due to the feedback from the task, enhancing signal transmission to M1 (Ohashi et al. 2019). The enhanced signal transmission from the somatosensory cortex increases the efficiency of signal integration in M1, therefore reducing the delay of the final output signal being sent. Transcranial direct current stimulation (tDCS), a subthreshold brain stimulation technique, has been shown to alter the excitability of internally activated M1 neurons, thereby making them more responsive to inputs from other brain regions. When tDCS is applied to M1, the increased rate of M1 output results in a robust learning effect, particularly during the early stages of acquiring and consolidating a simple motor task (Alonzo et al. 2012; Floyd et al. 2020; Pellicciari et al. 2013).

The cerebellum is an adaptive controller that aids in the error correction of motor tasks (Warthen et al. 2024). Through a large number of interconnections, the cerebellum can receive and integrate afferent signals from different cortical and subcortical regions (Habas 2021). Coordination between the cerebellum and higher/lower processing centers, via complex inhibitory Purkinje cells, permits further signal interpretation and distribution, particularly with M1 (Buckner et al. 2011; Luft and Buitrago 2005). The cerebellum has an influential role during the early stages of motor learning, when there is the greatest amount of error and task refinement takes place (Rurak et al. 2022). This has made the cerebellum an additional site of interest for tDCS stimulation (Grimaldi et al. 2016). Application of tDCS over the cerebellum alone during various motor learning tasks has yielded mixed results (Warthen et al. 2024).

It is recognized that during motor learning of a bimanual task, there is significant communication taking place between M1 and the cerebellum (Spampinato and Celnik 2017; Spampinato et al. 2017). Connections between M1 and the cerebellum along the cerebello‐thalamo‐cortical (CTC) pathway and cortico‐ponto‐cerebellar (CPC) tract (Allen and Tsukahara 1974; Dum and Strick 2003; Evrard and Craig 2008) modulate error correction and are important for feedback and feedforward control of complex motor tasks (Pisotta and Molinari 2014; Shadmehr and Krakauer 2008). Previous research by Liebrand et al. (2020) has identified that these pathways can potentially be influenced by cerebellar tDCS, but the results appear to be inconsistent. For example, previous studies applying anodal cerebellar tDCS during a sequence learning task have observed a beneficial effect of stimulation (Ferrucci et al. 2013; Shimizu et al. 2017). However, other studies also applying anodal cerebellar tDCS to a sequence learning task saw no beneficial effect of stimulation, possibly indicating a complex internal relationship between the demands of the task and the optimal tDCS configuration (Ballard et al. 2019; Guimaraes et al. 2023; Nguemeni et al. 2021).

Electrical stimulation variables such as current, polarity, electrode number, and placement (Naros et al. 2016; Stagg et al. 2011; Woods et al. 2016) can all be manipulated within a tDCS arrangement in different ways, producing different effects on learning (Guimaraes et al. 2023). There is a non‐linear dose‐response relationship between tDCS stimulation intensity and learning outcomes (Khalil et al. 2023), as increasing tDCS intensity can have a greater cortical effect, cause a reversal of polarity‐dependent effects, or cause a plateau (ceiling effects) (Guimaraes et al. 2023; Woods et al. 2016). Studies have yielded mixed results when assessing the effect of motor learning with tDCS set at different intensities (between 0.2 mA and 3.0 mA), suggesting optimal intensity parameters for a given task have yet to be standardized (Agboada et al. 2020; Chew et al. 2015; Mosayebi Samani et al. 2019). Furuya et al. (2014) proposed experience as a confounding characteristic also contributing to these effects. During their study, comparing untrained and highly trained individuals, they observed positive learning results in the untrained group following both anodal and cathodal stimulation: both finger‐key contact duration and its variability during keystrokes, as well as the variability of the inter‐keystroke interval, improved. However, the highly trained group did not produce the same improvement in their finger key contact duration and, in fact, increased their variability in the hand contralateral to the anode. Highly trained individuals, through practice, will have already gone through the initial stages of motor learning and increased synaptic plasticity, which may possibly void the effectiveness of tDCS (Bonfanti and Charvet 2021; Guimaraes et al. 2023).

It is generally agreed that anodal tDCS yields excitatory depolarizing effects on a neuron's membrane potential, whereas the effects of cathodal tDCS are inhibitory and hyperpolarizing (Bruckner and Kammer 2017; Lang et al. 2004; Nitsche and Paulus 2000). Anodal and cathodal stimulation of M1 involves a low‐intensity electrical current traveling from a positive electrode (anode) through the scalp and brain and exiting via a negative electrode (cathode). During anodal stimulation, the positive electrode is over the target brain area, and during cathodal stimulation, the negative electrode is over the target brain area. The specific route of the stimulation taken between the electrodes is highly variable; however, in the case of anodal stimulation, it appears to significantly increase M1 excitability, synaptic activation, and long‐term potentiation (LTP), resulting in learning‐related increases in M1 activity (Jwa et al. 2022; Qi et al. 2022; Thair et al. 2017; K. Yang et al. 2021). The standard polarity dependence for M1 may not transfer to the cerebellum during bimanual motor tasks due to the remote inhibitory tone it projects onto M1 and other brain areas (Fernandez et al. 2018). The cerebellum has inhibitory Purkinje cell projections that collate into cerebellar nuclei, which can then facilitate or inhibit the functionality of other brain regions (Galea et al. 2009; Pope and Miall 2012). During simple, gross motor tasks, cathodal cerebellar tDCS has demonstrated inhibitory effects on the cerebellum and greater excitability in M1 (Herzfeld et al. 2014; Jayaram et al. 2012; Weightman et al. 2021), whereas anodal cerebellar tDCS has demonstrated excitatory effects in the cerebellum and M1 inhibition (Azarpaikan et al. 2021; Galea et al. 2011; Patel et al. 2019). However, with predominantly cognitive tasks these same effects are not always observed due to disinhibition of areas such as the dorsolateral prefrontal cortex, which are heavily involved in cognitive processing (Bast et al. 2017; Pope and Miall 2012). Removal of the inhibitory cerebellar input to this area could release cognitive resources of the working memory and improve efficiency of cognitive processing during the task (Grimaldi et al. 2016; Grinschgl et al. 2023). Utilizing a bimanual task that requires complex motor and cognitive processing demands a balance of facilitation and inhibition modulated by cerebellar pathways and could prevent the polarity‐dependent effects of cerebellar tDCS from being observed (Grimaldi et al. 2016).

There have been a limited number of studies investigating bimanual task learning using bilateral tDCS montages (Furuya et al. 2014; Furuya et al. 2013; Gomes‐Osman and Field‐Fote 2013; Greenwell et al. 2025; Greenwell et al. 2025; McCambridge et al. 2016; Pixa et al. 2017a, 2017b; Vancleef et al. 2016). Original work from our lab has demonstrated that 2 mA of a‐tDCS applied to the active hemisphere, paired with 2 mA of cathodal cerebellar tDCS, significantly improved performance in a unimanual, keyboard‐pressing task (Meek et al. 2019). We then went on to show small rates of improvement in a bimanual video game racing task with bilateral anodal tDCS of M1 at 1 mA (Greenwell et al. 2025b). This led to a subsequent investigation on the combined multifocal configuration of bilateral M1 anodal and cerebellar cathodal tDCS at 2 mA being used while performing a bimanual video game racing task (Greenwell et al. 2025a). Contrary to what was hypothesized, we found there was a significant decrease in performance by the group receiving active stimulation.

The purpose of the present study was to determine if the negative learning results we found previously were due to the polarity of the current delivered to the cerebellum, or if another mechanism such as a ceiling effect of current intensity could be responsible. We hypothesize that both current and intensity may have affected the previous learning results. The current study used two groups (group four: biM1a + CBa, 2 mA; *n* = 23, group three: biM1a + CBsham, 2 mA; *n* = 23) with a classic pre‐, practice‐ and post‐assessment design that included a retention assessment approximately 24 h later. Data for two more tDCS conditions (group two: biM1a + CBc, 2 mA; *n* = 20, group one: SHAM; *n* = 21) was taken from Greenwell et al. (2025). The significance of this study, and many others on tDCS, is determining the optimal parameters for stimulation in motor skill learning.

## 2 Methods

### 2.1 Participants

A total of 46 right‐handed, healthy young adults (18 female and 28 male), between the ages of 18 and 45 years, participated in the study. Participants reported no brain injury, neurological disorder, or skeletal muscle disorder in their upper extremities and were physically capable of playing a video game with a standard controller. Subjects were instructed to avoid any over‐the‐counter stimulants containing caffeine (coffee, soda, tea, energy drinks, pre‐workout supplements) or nicotine (tobacco, nicotine pouches, gums, etc.) for at least 12 h before each of their two visits. At the beginning of the first visit, subjects were given a copy of the informed consent, which was explained to them by the researchers. They were also given a screening questionnaire and a video game experience questionnaire and had their handedness determined using the Edinburgh Handedness Inventory (Oldfield 1971). The study was approved by the Indiana University Institutional Review Board and was conducted according to the Declaration of Helsinki.

The video game experience questionnaire was used to determine the age participants began playing video games, the average hours they played each day, what mode of gaming they were using (i.e., mouse and keyboard, controller, touch screen, etc.), and what genre of game they were playing (i.e., first‐person shooter, racing, role‐playing etc.).

### 2.2 Procedures

**FIGURE 1:**
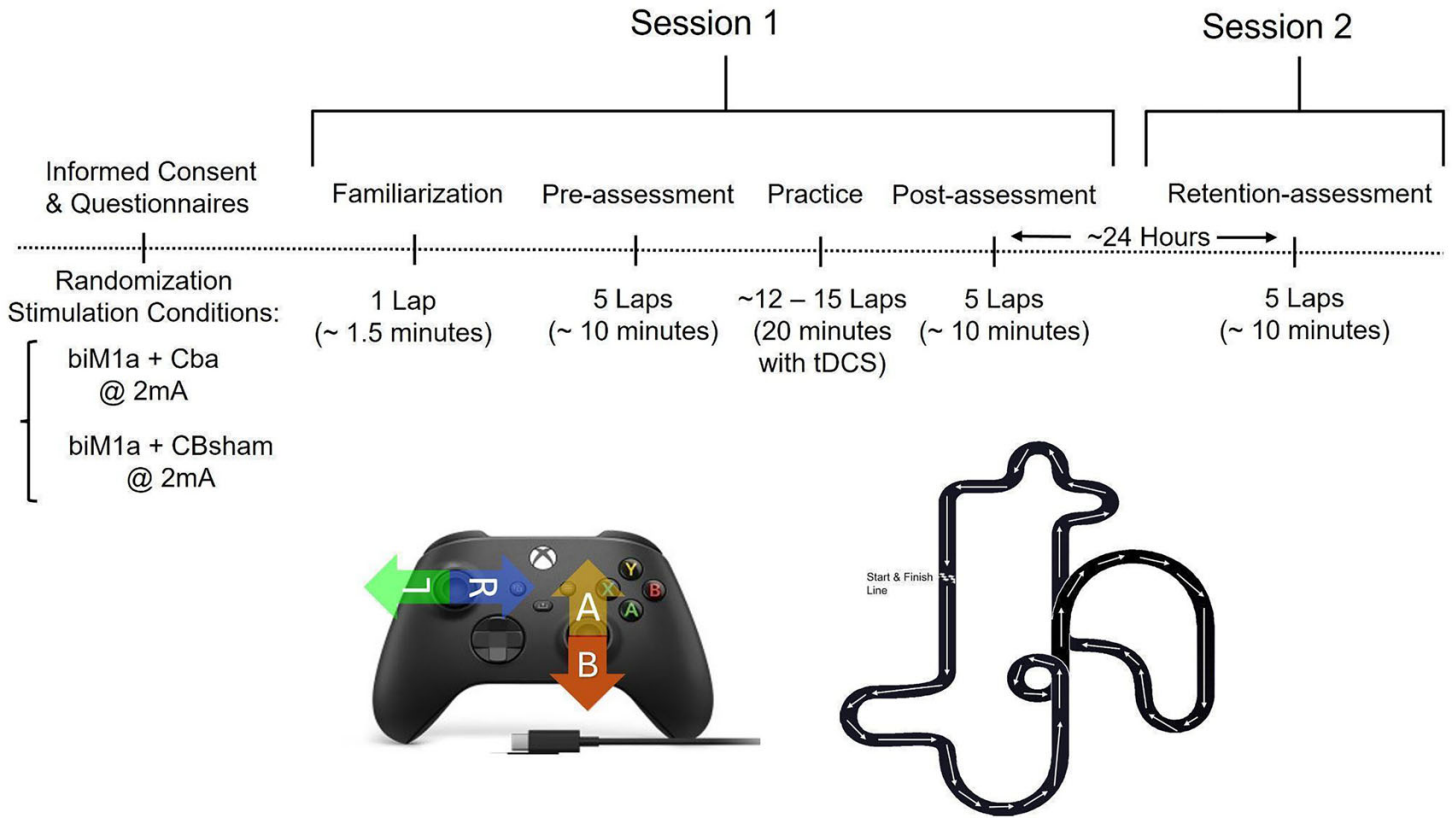
**(A)** Protocol timeline showing randomization, paper work, familiarization, assessment blocks and practice period and **(B)** Standard video game controller and controls used in the study and screenshot of the racing game track.

The study utilized a randomized, single‐blinded, between‐subjects study design with three active tDCS stimulation conditions. Subjects visited the lab for two testing sessions with ∼24 h between each visit. For the first visit, subjects completed the following procedures: (1) a one‐lap familiarization of the racing videogame, (2) five individual one‐lap races to serve as the pre‐test, (3) a 20 min practice period doing individual laps on the same track, and (4) five individual one‐lap races to serve as the post‐test. For the second visit, subjects only completed five individual one‐lap races on the same track for the retention assessment without any tDCS stimulation. (see Figures 1A, 1B).

### 2.3 Video Game Racing Task

Participants played an open‐source racing game (SuperTuxKart) during both of their visits. The game was played using a standard Microsoft Xbox One controller. Participants sat approximately 60 inches from the display monitor and had the controls explained to them before they began their familiarization lap: the left analog joystick steered left and right, and the right analog joystick used forward and backward motions to accelerate and brake, respectively. If the kart was standing still, pulling the right joystick back would make it go in reverse.

Participants were informed that their goal was to complete each lap as quickly as possible and to continuously focus on trying to improve on their previous scores. They were also instructed to lay the controller down on the desk between laps to rest/relax their hands and that a researcher would instruct them when to pick up the controller for their next lap (∼30 s). Controls were explained to the subjects, and they were given a single familiarization lap to test them without their time being recorded. Subjects were also informed that, aside from control reminders, researchers could not provide tips or advice on how to improve their performance and that control reminders would only be provided if subjects asked for them. In an effort to keep them focused on the task at hand, subjects were instructed by the researcher to “keep thinking of strategies for improvement” after every other lap.

After five one‐lap races of the pre‐assessment, participants completed a fixed 20 min (approximately 12–15 lap) practice phase while receiving tDCS stimulation. There was a 5‐ to 10‐min setup period, which is detailed in the tDCS Stimulation section below. At the conclusion of the 20 min, the participant was given approximately 5 to 10 min to focus on strategies for improvement while the tDCS headset was removed before completing five, one‐lap races as part of their post assessment. Five individual one‐lap races were also used in their retention assessment. In total, participants completed approximately 27–30 laps over their two visits.

The game's code was modified to make the karts faster, heavier, and harder to steer, increasing the difficulty of the task by reducing task familiarity. Subjects were only able to use the joysticks for control of the game (all other buttons were disabled), and all “power‐ups” and special features of the games were turned off. To reduce performance variability, the track used for this study was closed on all sides so that subjects could not leave the track or take shortcuts. However, since large wall collisions significantly slowed the kart down, optimal times were achieved through a combination of accurate steering with timely control of acceleration and braking through the curves.

### 2.4 tDCS Stimulation

Active and SHAM tDCS were delivered via three Soterix Medical 1 × 1 low‐intensity transcranial direct current stimulators during the practice phase. Participants were randomly sorted into two different stimulation groups, and data for two other tDCS conditions were included from Greenwell et al. (2025). The four conditions were (1) complete SHAM stimulation with electrodes bilaterally over M1 and over the cerebellum (*n* = 20), data from Greenwell et al. (2025), (2) bilateral, anodal M1 tDCS with cathodal cerebellar stimulation (biM1a + CBc, *n* = 21), data from Greenwell et al. (2025) (3) bilateral, anodal M1 tDCS with anodal cerebellar stimulation (biM1a + CBa, *n* = 23), (4) bilateral, anodal M1 tDCS with SHAM cerebellar stimulation (biM1a + CBSHAM, *n* = 23). All stimulation conditions used 2 mA of current for the entire 20‐minute practice duration.

At the end of the pre‐assessment all the participants had the hand region of M1 marked (C3 and C4 according to the 10‐20 EEG Coordinate System). Using a tape measure, the distance halfway between the nasion and occipital protuberance was identified. A second set of dots was made at the point halfway between the tragi. The point of intersection of these marks was noted as the vertex of the skull. M1 was located with measurements of 6 cm to the left and right of the vertex and 2 cm forward. After these points were marked on the subject, saline was applied to them, and a standard electrode configuration was added to the participant's head, similar to that of Greenwell et al. (2025). Using the Caputron Universal tDCS headstrap (Caputron, 2023), two 5 × 7 cm anodal electrodes were placed in saline‐soaked sponges over the marked hand regions of M1. Two cathodal 5 × 7 cm electrodes were also placed in saline‐soaked sponges on the participants' supraorbital region (Fp1 and Fp2 according to the 10‐20 EEG Coordinate System).

**FIGURE 2:**
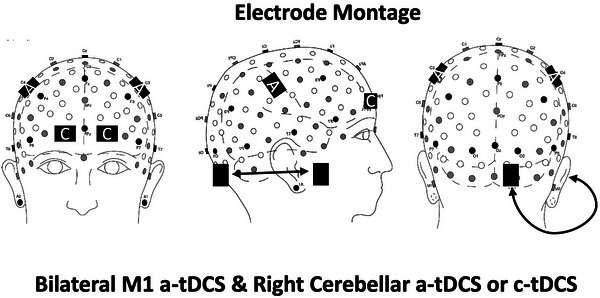
Diagram showing the electrode placement for the bilateral, anodal M1 tDCS stimulation and for the cerebellar tDCS stimulation conditions.

All participants had a second set of 3 × 5 cm electrodes on their head. The 3x5 cm anodal electrode was placed in a saline‐soaked sponge approximately 1 cm to the right of the inion (the highest part of the occipital protuberance), over the cerebellum. The second 3 × 5 cm electrode (cathodal) was placed in a saline‐soaked sponge, just above the buccinator muscle. The placement of the electrodes for the bilateral, anodal M1 tDCS and for the anodal/cathodal cerebellar tDCS is shown in Figure 2.

Once all electrodes were fixed, the stimulator was switched on and contact quality checked. After the check had finished, participants were asked if they were ready for the stimulation protocol to begin. The stimulators ramped up to 2 mA and operated at this intensity for 20 min during the practice phase. If the participants were a part of the biM1a + CBsham condition, the tDCS machine supplying the cerebellar electrodes would ramp up to 2 mA and then ramp back down to 0 mA, before the first lap of the practice phase was started.

The end of the practice phase was marked by the stimulator counting down its last minute of activity. Laps only counted during the practice phase if they were completed while receiving 2 mA of stimulation. After the countdown had reached 0 s, the stimulator ramped down its current to 0 mA and the headset was removed.

### 2.5 Data Analysis

The average of the five lap times for the pre‐test, post‐test, and retention was used to distinguish racing performance. Subjects were excluded from future analysis for demonstrating baseline performance pre‐test times more than 2.5 standard deviations above the mean times from the entire data set. The total number of participants included in each group is reflected at the start of the results section. In addition to absolute changes in lap times, the percent change in lap times was also calculated to normalize any differences in starting skill between groups. The practice period was analyzed differently because the number of laps completed during the 20 min practice stage varied among subjects; depending on how quickly they completed each lap and the subsequent 30 s rest period, approximately 12–15 laps were completed. The average of the first and last two practice laps was used from that period. Finally, the total number of hours per week subjects spent playing any type of video games was collected from the gaming experience questionnaire filled out at the beginning of the study.

### 2.6 Statistics

A two‐way repeated measures ANOVA was used to compare within‐day and between‐day changes in lap times (in seconds) across the four stimulation conditions (SHAM, biM1a + CBc, biM1a + CBa, biM1a + CBsham). The same comparison was made using gaming experience as a covariate in an ANCOVA. These tests were repeated on the percent change in lap time in order to normalize the starting point for all subjects. A bivariate correlation was used to directly compare gaming experience and the improvements in lap times. Bonferroni post‐hoc tests were used when appropriate for multiple comparisons. Effect sizes were calculated as partial eta squared (*ηp^2^*), and a *p*‐value of 0.05 was considered statistically significant. All analysis was performed in SPSS 29.

## 3 Results

**FIGURE 3:**
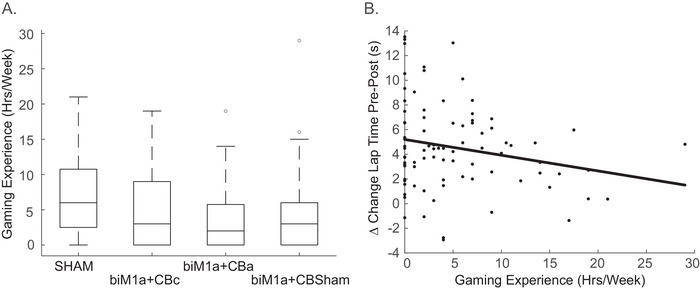
**(A)** Boxplot displaying the average gaming experience for each stimulation condition and **(B)** Scatterplot representing the significant negative correlation for the within day change in lap time and gaming experience (*r* = −0.224, *p* = 0.036).

Gaming experience alone was not different between groups (*F* (3, 83) = 0.889, *p* = 0.450, partial *ηp^2^* = 0.031, Figure 3A), so any differences observed were not due to any one group being full of gamers or non‐gamers. A significant negative correlation was found between gaming experience and the change in pre‐post lap times within a single day of practice across all subjects (*r* = −0.224*, p* = 0.036, Figure 3B), showing that the less experience you have with gaming, the greater the improvement regardless of stimulation.

**FIGURE 4:**
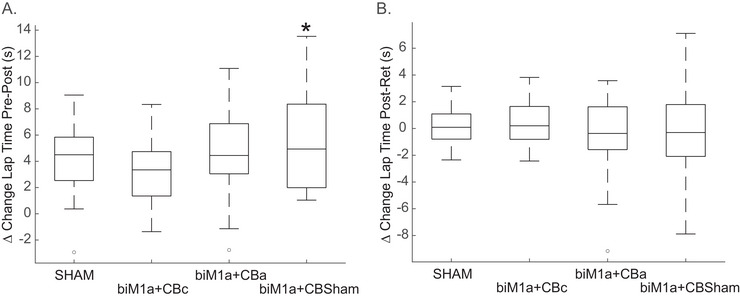
**(A)** Boxplot displaying the absolute change in lap time (seconds) from the pre‐post test in session 1, where Condition 4 (biM1a + CBSHAM) had the greatest improvement. *represents *p* < 0.05 and **(B)** Boxplot displaying the absolute change in lap time (seconds) from the post‐test in session 1 to the retention session 2. There were no significant differences.

Due to this negative correlation, we used gaming experience as a covariate in an ANCOVA and found that there was a significant difference across stimulation conditions pre‐post within the same day (*F* (3, 83) = 2.957, *p* = 0.025, partial *ηp^2^* = 0.126, Figure 4A), with the largest improvement in lap time being in the biM1a+CBsham group (6.05 ± 4.28 s). However, the same ANCOVA performed on the difference between days, or the post‐test in day 1 and the retention in day 2, displayed no differences across stimulation conditions (*F* (3, 83) = 0.238, *p* = 0.87, partial *ηp^2^* = 0.020, Figure 4B). When gaming experience was not used as a covariate, and an ANOVA was performed on the same data, there were no differences within (*p* = 0.063) or between days (*p* = 0.797).

**FIGURE 5:**
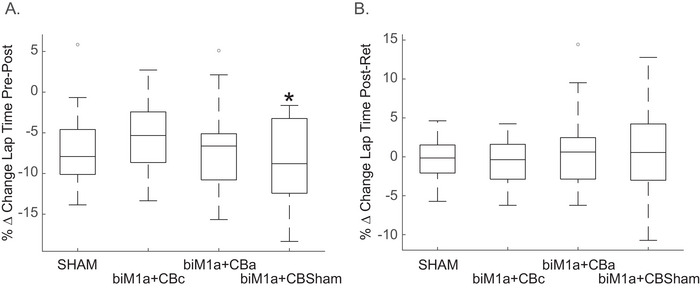
**(A)** Boxplot showing the % change in lap time from the pre‐post test in session 1, where Condition 4 (biM1a + CBSHAM) had the greatest improvement. *represents *p* < 0.05 and **(B)** Boxplot showing % change in lap time from the post‐test in Session 1 to the retention session 2. There were no significant differences.

**FIGURE 6:**
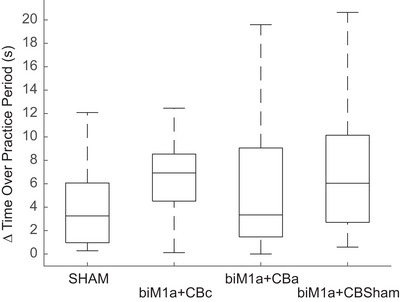
(A) Boxplot showing the absolute change in lap times (s) from the beginning of the practice session to the end of the practice session. There were no significant differences.

One potential issue with the data was that race times in the pre‐test were significantly different across the four stimulation conditions (*F* (3, 83) = 6.399, *p* < 0.001, partial *ηp^2^* = 0.188), with the biM1a+CBc group having significantly faster pre‐test lap times than the biM1a + CBa or biM1a + CBsham groups (*F* (3, 83) = 5.024, *p* = 0.003; (3, 83) = 3.641, *p* = 0.016, respectively). Because of this, we also ran the same ANCOVA analysis on the percent change in lap times to normalize the differences in the pretest across groups. Gaming experience was again used as a covariate in an ANCOVA, and there was still a significant difference across stimulation conditions pre‐post within the same day (*F* (3, 83) = 2.782, *p* = 0.032, partial *ηp^2^* = 0.119, Figure 5A), with the largest percent change in lap time being in the biM1a + CBsham group (−8.98 ± 5.55%). There were still no differences between days in the percent change in lap times (*F* (3, 83) = 0.314, *p* = 0.815, partial *ηp^2^* = 0.018, Figure 5B). Figure 6.

Data from the practice periods was variable, as subjects completed anywhere from 12 to 15 laps during the 20 min, depending on how fast they completed each lap and allowing for a 30 s rest period between laps. Lap times were averaged for the first and last two laps during the practice period, and the percent change was calculated. There was a trend for a significant difference between stimulation conditions (*F* (3, 84) = 1.98, *p* = 0.11, Figure 6), with the percent improvement being the largest in the biM1a + CBsham group (7.2 ± 5.27%).

## 4 Discussion

The main finding of this study was that bilateral M1 anodal tDCS results in the best stimulation condition when playing a racing video game using dual joysticks as controls. The most significant of these effects were observed during the within‐day session and were largely dependent on the gaming experience of the individual, as those that do not consistently play video games will experience the largest learning improvement with this stimulation paradigm. Despite previous literature suggesting a key role of the cerebellum in successfully completing tasks like these, we did not find any advantage to using either anodal or cathodal cerebellar stimulation when paired with bilateral M1 tDCS. These findings are consistent with the conclusion that the effectiveness of tDCS is entirely dependent on the tasks studied and the sites stimulated.

Consistent with the current literature, M1 appeared to be an effective stimulation site for accelerating learning. We found the group (biM1a + CBsham) with active stimulation only applied to M1 yielded the greatest within‐day learning effects. Our results were consistent with many previous studies showing the effectiveness of M1 stimulation on motor learning (Fujimoto et al. 2014; Goodwill et al. 2013; Karok and Witney 2013; Keitel et al. 2018; Pantovic et al. 2023; Reis et al. 2009; Stagg et al. 2011; Vines et al. 2008), primarily online learning within a single session. Bimanual movements typically require increased motor activation between the hemispheres. and previous studies have also observed accelerated online motor learning outcomes using bilateral M1 tDCS paired with a bimanual task (Gomes‐Osman and Field‐Fote 2013; Greenwell et al. 2025; Lee et al. 2023; Pixa et al. 2017a, 2017b). Proficient control and coordination of both hands independently is important for the successful execution of a bimanual task; therefore, during online learning, bilateral tDCS may regulate the facilitatory effects in M1 of the dominant hemisphere while also modulating the interhemispheric inhibition from the non‐dominant hemisphere (Lindenberg et al. 2013; Williams et al. 2010).

While bilateral tDCS of M1 has shown accelerated bimanual task learning, there have been cases where learning has not been significantly accelerated, and additional factors have been proposed as influencing this result. Pixa et al. (2017a, 2017b) recognized that matching the tDCS montage and chosen bimanual task is important, as different levels of processing and possibly different cortical regions may be more or less involved depending on the task, and this may alter the efficacy of the chosen tDCS montage on learning (Naros et al. 2016; Stagg et al. 2018; Woods et al. 2016). Individual differences in stimulation sensitivity may also result in different online learning effects of bilateral M1 tDCS. Whether it is the curvature of the skull or the ability to excite an individual's internally activated neurons, subjects seem to have a fluctuating response to tDCS application. Future studies should investigate personalized tDCS protocols where there is a task‐intensity‐sensitivity match, which could potentially produce a more robust learning effect of multifocal tDCS (Chaieb et al. 2008; Manenti et al. 2013; Thair et al. 2017).

Previous work from our lab had demonstrated the potential benefit of combining cerebellar and bilateral M1 tDCS (Meek et al. 2019). During the current study, we analyzed several tDCS conditions, and when comparing the two groups receiving cerebellar tDCS along with bilateral M1 tDCS, the cerebellar anodal group had a greater within‐day decrease in lap time, but it was not significant. The cerebellum has inhibitory Purkinje cells that project to M1; it is believed that these connections can be inhibited by cathodal tDCS, resulting in a disinhibition of M1, and therefore, a greater excitatory effect could possibly be observed in M1 during motor learning (Allen and Tsukahara 1974; Galea et al. 2009; Galea et al. 2011; Weightman et al. 2021). However, the additive effects of the additional cerebellar stimulation sites and stimulation provided to M1 could be potentially increasing neural noise and possibly introducing noisy background input to M1, in turn decreasing the efficiency at which M1 is able to produce an output signal (Bonaiuto and Bestmann 2015). Anodal tDCS induces excitatory effects, and therefore, when placed on the cerebellum, could in theory strengthen the inhibitory signal projected from the cerebellum to M1 (Allen and Tsukahara 1974; Galea et al. 2009; Galea et al. 2011; Weightman et al. 2021). In less trained individuals this could hinder learning, as increased excitatory activity in M1 has been observed with motor learning (Hosp and Luft 2013). However, because of the increased bimanual use of the thumbs in activities of daily living (ADL), the cortical representation and, therefore, baseline motor control of these digits could be developed to a far enough extent that performance of a novel task may require more processing through brain regions for higher processing, such as the supplementary motor area and premotor cortex.

During the current study participants performed a bimanual dual joystick control task. The controls consisted of the left analog stick dictating steering and the right analog stick controlling the acceleration and deceleration of the kart. The control scheme became less dexterous in nature, as participants could continuously press the right analog stick forward, and in exchange for the kart travelling at full speed, a relatively small error would be produced if they were to hit the side of the kart on a barrier. Meaning, the need for persistent modulatory activity in the right thumb may have decreased. We have performed previous studies using a different track with no barriers so that participants could deviate from the course (Greenwell et al. 2025). This meant that to achieve a faster lap time, the thumb may have needed to be more actively engaged in mediating the speed of the kart during the trials so that the kart stayed on the track. However, because the previous track was not enclosed, there was high variability in race times that did not directly relate to the skill level of the subject. To create a more controlled environment in this study, we selected a track with barriers on the sides so that the entire course was enclosed. This vastly decreased the variability in lap time; however, it may have also removed the need for continuous error correction while playing. The reduced variability and unexpected absence of the need for continuous error correction could have had a bearing on the cerebellum's activity and contribution to the results of this study. We know the cerebellum has a key role in the error correction of motor tasks, and due to the chosen course and the number of trials, subjects completed numerous laps of the same track, perhaps reducing the overall variability and cerebellar activity during the task (Gluck et al. 2001; Herzfeld et al. 2018; Popa and Ebner 2018; Popa et al. 2016). Thus, it is more likely that the learning effects observed primarily took place in M1 and were the catalyst of improved performance.

Gaming experience proved to be an important factor in the current study. A significant negative correlation was found between gaming experience and the change in lap times from the pre‐assessment to the post‐assessment (within‐day changes) across all subjects. While experience was not significantly different between groups, less experienced video game players produced slower race times in the pre‐assessment, making their data more variable, contributing to significantly different pre‐assessment times across the four stimulation conditions. Previous research has observed novice learners' increased susceptibility to plastic changes and brain reorganization associated with learning (Bullard et al. 2011; Chang 2014; Kolb and Whishaw 1998). Experts have been observed to make less improvement in previous tDCS studies, demonstrating a possible ceiling effect. One proposed mechanism of tDCS is to increase the synaptic plasticity between neurons and induce long‐term potentiation effects; however, highly trained individuals, such as professional golfers and violinists, already have heightened‐focused cerebral activation (Lotze et al. 2003; Milton et al. 2007). Also, important to note is the possibility of generally higher synaptic plasticity within the thumb region of M1. Smartphone use, in particular messaging and scrolling on smartphones, has become a repetitive motor task performed, at times, for hours daily. Other studies have shown a functional enlargement of the hand region in professional musicians and racquetball players due to focused activation from practicing a motor task (Amunts et al. 1997; Pearce et al. 2000). It is plausible that cell phone use could mimic the effect of practice and induce these same cortical adaptations. Overall, this may reduce the efficacy of tDCS when applied to the hand region of M1 during a bimanual video game task, particularly when the use of both thumbs is required on the joysticks of the controller. To observe the effects of learning in the future and strengthen group comparability, it may be advantageous to use stratified randomization to sort the participants into their trial conditions based on gaming experience and try to recruit subjects who do not have a phone or play video games, though that may not be feasible.

Although this study was focused on the combined effects of bilateral M1 and cerebellar tDCS, other brain regions may have a significant role in motor learning and could potentially benefit from the application of tDCS. The supplementary motor area (SMA) and premotor cortex (PMC) influence the activity in M1 through direct and indirect pathways (Trajkovic et al. 2023). The cortico‐cortical pathway between the ventral premotor cortex is one of the most direct connections to M1 and ensures there is sufficient initiation of the preparatory steps needed for the execution of internally generated patterns of movement (Dum and Strick 2005). The supplementary motor area has been traditionally associated with the initiation of externally generated bimanual movements (Swinnen and Gooijers 2015). Single cell recording techniques in primates have indicated a sub‐pool of neurons that may be uniquely activated during the production of bimanual movements (Donchin et al. 1998). Mapping of the supplementary motor area following consistent grasping task execution in monkeys revealed an encroachment of the representative map for grasping over the areas of the other digits (Byl et al. 1996). This could in theory be applicable to the example of 2023 phone texting/scrolling and the ceiling effects observed in bimanual tasks using the thumbs to manipulate the controls. Future studies could expand on this theory to investigate the cortical maps participants start learning experiments with and compare them to maps taken after a practice period in order to monitor changes with practice and tDCS to provide further insight on the mechanisms that may limit the effects of tDCS.

The consolidation effects (between‐day changes) were relatively small compared to the within‐day changes, with the biM1a + CBc group exhibiting the largest between‐day change. The consolidation of a skill typically begins after practice and is influenced by sensory feedback and the internal model of the skill within the working memory (Luft and Buitrago 2005; Lugassy et al. 2018). In contrast to our protocol, Di et al. () performed nine sessions of anodal tDCS between a pre‐ and post‐assessment, and subsequently reported a facilitatory effect of tDCS on the offline, early consolidation of a visual perceptual learning task. For future studies, this demonstrates the potential benefit of additional tDCS blocks across many days to evaluate the real timeline of motor skill learning. Increasing the number of subjects would not have yielded different results besides slightly reducing the variability in retention session lap times, which were less than 1 s different between conditions. Both M1 and the cerebellum work together to transition the repetition‐dependent plasticity of M1 to the consolidation effects of the cerebellum, though only two sessions separated by ∼24 h were insufficient to evoke these changes (Breton and Robertson 2014; Hamel et al. 2017).

## 5 Conclusion

This study sought to investigate the polarity and current effects of cerebellar and bilateral M1 tDCS on the learning of a bimanual video game racing task. Utilizing a number of tDCS conditions, we were not able to observe any significant learning effects that differed from the current literature. Our findings confirmed that bilateral tDCS applied to M1 contributes to accelerated, within‐day learning and appears to be task‐specific and susceptible to ceiling effects of experience. While largely exploratory, our study highlights the need for further research into personalized tDCS montages that consider the specific demands of the task being performed and individual differences and experience levels of the participant. Future studies into video game use and tDCS should focus on the clinical application of tDCS to investigate if it can be used to help neurologically impaired gamers relearn how to play and the transferability of the bimanual relearning to other tasks.

## Author Contributions

**Quinn McCallion**: conceptualization, methodology, formal analysis, investigation, writing, data curation, and project administration. **Davin Greenwell**: conceptualization, methodology, formal analysis, investigation, writing, data curation, and project administration. **Bryan S Barry**: methodology, formal analysis, investigation, writing, and data curation. **Emelia E Duchow**: formal Analysis, investigation and writing; **Brach Poston**: methodology, writing, data curation, and project administration. **Zachary A Riley**: conceptualization, methodology, formal analysis, investigation, writing, data curation and project administration.

## Funding

The authors have nothing to report.

## Ethics Statement

The study was approved by the Indiana University Institutional Review Board (#22735) and was conducted according to the Declaration of Helsinki.

## Conflicts of Interest

The authors declare no conflicts of interest.

## Data Availability

The data that support the findings of this study are available from the corresponding author upon reasonable request.
